# Comparison of Effectiveness Between Cysticidal and Surgical Treatments in Patients with Intraventricular Neurocysticercosis: A Single-Center Experience

**DOI:** 10.3390/pathogens15010108

**Published:** 2026-01-19

**Authors:** Alex Aarón Agallo-Martínez, Rebeca Ramírez-Bustamante, Polet Juárez-Ortíz, Ximena Gutiérrez-Bonilla, Sergio Moreno Jiménez, Roger Carrillo-Mezo, Agnès Fleury

**Affiliations:** 1 Unidad de Neuroinflamación, Departamento de Medicina Genómica y Toxicología Ambiental, Instituto de Investigación Biomédicas, Universidad Nacional Autónoma de México, Instituto Nacional de Neurología y Neurocirugía Manuel Velasco Suárez (INNN-MVS), México City 14269, Mexico; alexaagallo@gmail.com (A.A.A.-M.); reberamirez65@gmail.com (R.R.-B.); poletjo15@gmail.com (P.J.-O.); ximenagbonilla1@gmail.com (X.G.-B.); 2 Departamento de Neurocirugía, Instituto Nacional de Neurología y Neurocirugía Manuel Velasco Suárez, México City 14269, Mexico; radioneurocirugia@gmail.com; 3 Departamento de Neuroimagen, Instituto Nacional de Neurología y Neurocirugía Manuel Velasco Suárez, México City 14269, Mexico; mayroger@hotmail.com; 4 Clínica de Neurocisticercosis, Instituto Nacional de Neurología y Neurocirugía Manuel Velasco Suárez, México City 14269, Mexico

**Keywords:** neurocysticercosis, ventricle, *T. solium*, cysticidal treatment, neuroendoscopy, functionality, Mexico

## Abstract

Neurocysticercosis (NCC) remains a major public health problem in endemic countries. Clinical manifestations and therapeutic strategies vary depending on the location of the parasite. While the benefits of cysticidal treatment are well established for parenchymal and subarachnoid NCC, the optimal management of intraventricular NCC (IVNCC) remains controversial. We conducted a retrospective study of 51 patients: 37 (72.54%) received cysticidal treatment as initial therapy and 14 (27.45%) underwent neurosurgical intervention. Although six months after treatment, the proportion of patients with inactive disease was higher in the surgical group, no significant difference was observed after one year. Patients in both groups showed significant improvement in functionality as measured by the Karnofsky Index (KI), with no significant difference between groups. These results are consistent with cysticidal treatment being a valid therapeutic option for IVNCC, with the choice of management largely determined by the available medical infrastructure and the degree of specialization of healthcare personnel.

## 1. Introduction

Neurocysticercosis (NCC), caused by the localization of larvae of the *Taenia solium* parasite in the central nervous system, is a frequent and potentially very serious parasitic disease [1]. Although its incidence is declining in several countries [2], it remains a public health problem in endemic countries in Latin America, Asia, and Africa. The life cycle of *Taenia solium* can only be maintained if pigs have access to human excrement, which is generally found in rural areas where pigs are raised freely, and defecation occurs in the open air. The presence of the parasite is therefore considered a sentinel indicator of poverty and poor sanitation.

The clinical presentation, severity, prognosis, and treatment depend on the compartment where the parasites are found [2].

There are different therapeutic approaches for neurocysticercosis [3]. Since the 1980s [4,5], the demonstration of the efficacy of two drugs (albendazole, ABZ, and praziquantel, PZQ) has completely changed the therapeutic outlook, whereas previously the only treatment was surgical. For parenchymal localizations, cysticidal drugs (ABZ and PZQ) have proven effective in destroying the parasites and, although their use was initially questioned, they are now considered the first-line treatment for most patients with this form of the disease [6]. For the subarachnoid form, cysticidal treatment after correction of intracranial hypertension (ICH) if necessary is also accepted, although two modifications have been suggested: prolonged administration [7] or a higher dose [8] for 10 days, repeated several times every 6 months if necessary to achieve destruction of all parasites [9]. In the case of ventricular infection, the latest recommendations favor surgical rather than medical management [6], although published case series have demonstrated the effectiveness of both types of treatment, surgical and medical [8,10,11,12,13]. This ongoing controversy persists largely because the available evidence is derived mainly from small case series, often emphasizing technical success rather than long-term clinical or functional outcomes, and rarely providing direct comparisons between medical and surgical strategies. Moreover, data from endemic, resource-limited settings, where access to specialized neurosurgical care may be variable, remain scarce.

To help fill these gaps, we conducted a retrospective comparative study evaluating cysticidal therapy and neurosurgical management in patients with intraventricular NCC, with a specific focus on disease activity, functional outcomes, and the need for complementary management of intracranial hypertension.

## 2. Materials and Methods

We conducted a retrospective study including patients diagnosed with ventricular NCC at the Instituto Nacional de Neurología y Neurocirugía Manuel Velasco Suárez (INNNMVS) after 2000. The INNNMVS is a tertiary referral center equipped with the most advanced diagnostic tools in Mexico. As a result, the INNNMVS attracts severely affected patients, particularly those with intraventricular localization of the parasite. Although it accepts patients without social security from all over Mexico, most of the patients treated are from central Mexico.

Eligibility criteria are summarized in Table 1. Briefly, adult patients (≥18 years) with a definitive diagnosis of intraventricular NCC according to accepted criteria [14,15], complete clinical and radiological records, and follow-up until disease inactivity were included. Patients with incomplete data, lack of definitive diagnosis, or loss to follow-up before parasite resolution were excluded.

**Figure 1:**
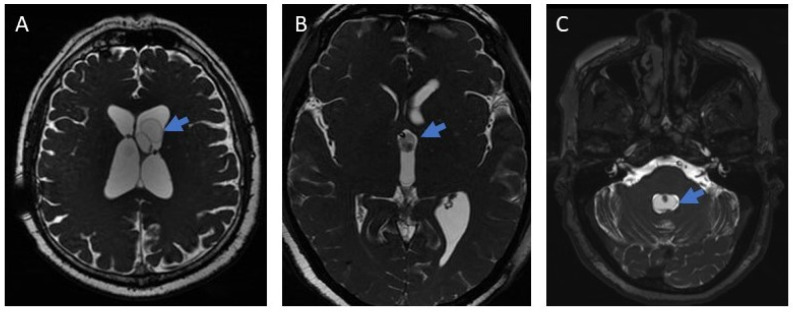
Images of ventricular neurocysticercosis (3D MRI). (**A**): Lateral ventricle; (**B**): Third ventricle; (**C**): Fourth ventricle. The arrows indicate the parasite in each figure.

The diagnosis of ventricular localization of the parasites was made by an expert neuroradiologist based on magnetic resonance imaging (MRI, T1- and T2-weighted sequences, SPGR, FLAIR, and 3D T2-weighted sequences) showing cystic lesions with the characteristics of cysticerci [16] (Figure 1). In cases of surgical extraction, the histopathological report had to confirm the diagnosis.

Patients were divided into two groups based on the initial treatment received: cysticidal drugs or surgical management.

The pharmacological treatment regimens and timing of administration are summarized in Table 2.

Albendazole and praziquantel were administered using commercially available formulations (Eskazole^®^/Zentel^®^ for albendazole and Cisticid^®^ for praziquantel), with manufacturers depending on institutional availability. Dexamethasone was administered as a standard commercial preparation. Albendazole was prescribed at a dose of 30 mg/kg/day, based on prior experience at our institution and supported by a previously published study showing improved efficacy compared with the conventional dose of 15 mg/kg/day [8].

Surgical treatment most often consisted of endoscopic removal of the parasites. In only two cases, a suboccipital craniotomy was performed to remove the parasites.

In cases of intracranial hypertension requiring surgical relief, two procedures were used: the placement of a ventriculoperitoneal shunt (VPS), a commonly performed, low-complexity neurosurgical procedure, or an endoscopic third ventriculostomy, performed during the same surgical session as endoscopic surgery.

Patients were followed until radiological evidence of disease inactivity was achieved, confirmed by at least two MRI scans in cases where residual activity was uncertain. Follow-up duration varied according to clinico-radiological features and patient availability, ranging from 5 to 164 months. Given the retrospective nature of the study and the long inclusion period, follow-up duration was heterogeneous, and no predefined monitoring schedule was applied.

A database was created containing demographic, clinical, and radiological information at the time of diagnosis, the type of treatment applied, and clinical and radiological progression during follow-up. Functionality was assessed at the time of diagnosis and during disease inactivity using the Karnofsky Index (KI), which measures patients’ ability to perform normal activities. The KI ranges from 100% (normal condition and full functionality) to 0% (death); it was first described in the field of oncology [17] and then adapted to various medical scenarios [18,19].

### Statistical Analysis

For descriptive purposes, continuous data were summarized as arithmetic means with standard deviation (SD). Differences between continuous variables were assessed using Student’s *t* test, Mann–Whitney U test, or Wilcoxon matched-pairs signed-rank test, as appropriate, according to data distribution (assessed using Kolmogorov–Smirnov test) and whether observations were paired. Categorical variables were analyzed using Fisher’s exact test or the chi-square test. All analyses and graphical representations were performed using GraphPad Prism software (version 8.0.2).

## 3. Results

### 3.1. Study Population and Baseline Characteristics

**Figure 2:**
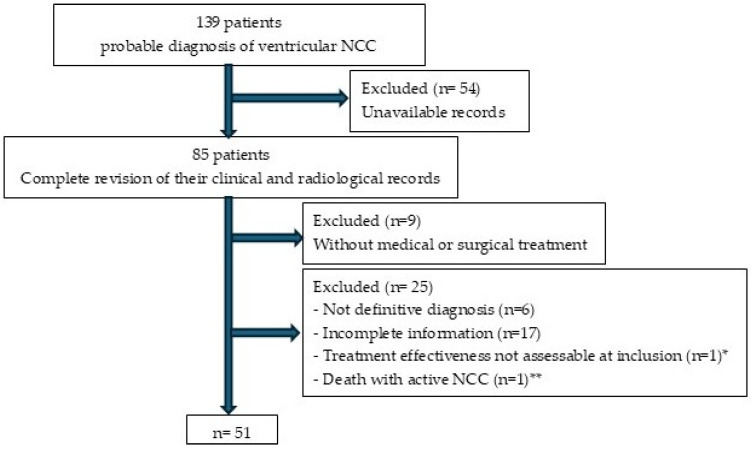
Patient enrollment flowchart for the study. * At the time of this study, the patient had received cysticidal treatment less than 6 months earlier. ** Patient who underwent surgical treatment and died from surgical complications before the parasites became inactive.

We initially reviewed 139 clinical records. However, as shown in Figure 2, only 51 patients meeting the inclusion criteria were ultimately considered.

We divided the patients into two groups based on the initial treatment received: cysticidal drugs (n = 37) and surgical management (n = 14). As shown in Table 3, the two groups did not differ significantly in terms of demographic, clinical, or imaging characteristics at the time of diagnosis. In both groups, most patients had intracranial hypertension (ICH), with vesicular parasites located in the fourth ventricle. Most also had parasites in other areas of the brain, mainly in the subarachnoid space (basal cisterns) in a vesicular state. It should be noted that no patients in either group had extraneural cysticercosis.

It should be noted that none of the patients developed classic Bruns syndrome, which is characterized by episodes of headache, dizziness, and vomiting triggered by sudden head movements and is usually associated with intraventricular NCC [20].

### 3.2. Initial Treatment Strategies

As summarized in Table 4, initial treatment in the cysticidal group consisted of a 10-day course of ABZ combined with corticosteroids. In the surgical group, parasite extraction was performed endoscopically in 12 patients and by suboccipital craniotomy in 2 patients. In addition, five patients received a course of ABZ immediately after surgery due to incomplete extraction of the parasites.

This initial treatment resulted in disease inactivation (i.e., destruction of all ventricular parasites) in 25 patients in the cysticidal group (67.56%) and in 11 patients (78.57%) in the surgical group, with no significant difference between the two groups (*p* = 0.51) (Table 4). However, despite the inactivity of their disease, 9 patients in the cysticidal group and 1 patient in the surgical group experienced VPS dysfunction and required additional surgery (revision or replacement of the VPS) to control intracranial hypertension.

### 3.3. Additional Treatments to Reach Disease Inactivity

Additional treatments aimed at achieving disease inactivity were administered to 15 patients. As detailed in Table 4, these treatments consisted mainly of additional cysticidal cycles, one cycle in 10 patients (9 in the cysticidal group and one in the surgical group) and two cycles in 5 patients (3 in the cysticidal group and two in the surgical group). Additional endoscopic cyst removal was performed in only one patient, in the cysticidal group. Seven patients also required additional management for ICH, in all cases with a VPS, six of whom had never had ICH before. Fourteen patients in the cystic group (37.8%) did not require surgical management of intracranial hypertension. In this group, the presence of parasites in the lateral ventricles was higher (7/14, 50%) than in the group requiring surgical management of ICH (4/23, 17.4%) (*p* = 0.06).

### 3.4. Surgical Management of ICH

With regard to the surgical management of ICH (Table 4 and Table 5), in the cysticidal group, a VPS was placed in 17 patients (45.94%) prior to cysticidal treatment. In the surgical group, ICH management was initially performed in all patients, with third ventriculostomy in 8 of them, VPS in 4, or both procedures in 2. These procedures were generally performed during the same surgery as the extraction.

### 3.5. Final Outcomes and Functional Status

**Figure 3:**
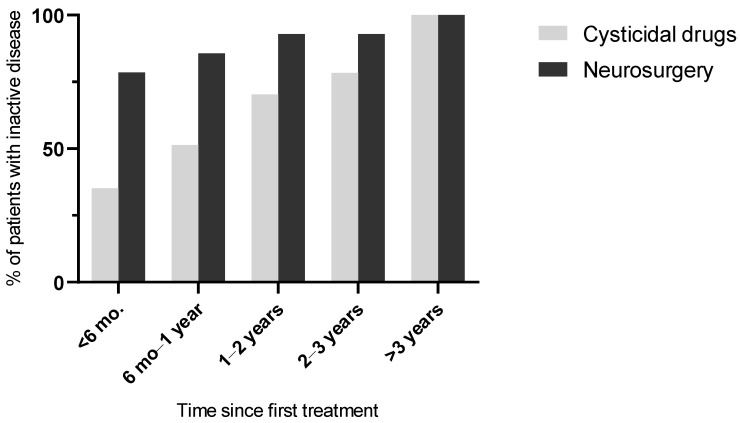
Cumulative proportion of patients achieving neurocysticercosis inactivity over time according to initial treatment strategy. Data are derived from the follow-up assessments summarized in Table 4.

At the end of follow-up (Table 6), the disease was inactive in all patients. At 6 months post initial treatment, the proportion of patients with inactive disease was significantly higher in the surgical group than in the cysticidal group (*p* = 0.010), but after this period, this proportion was similar between the two groups. The cumulative evolution of disease inactivity according to treatment strategy is illustrated in Figure 3.

**Figure 4:**
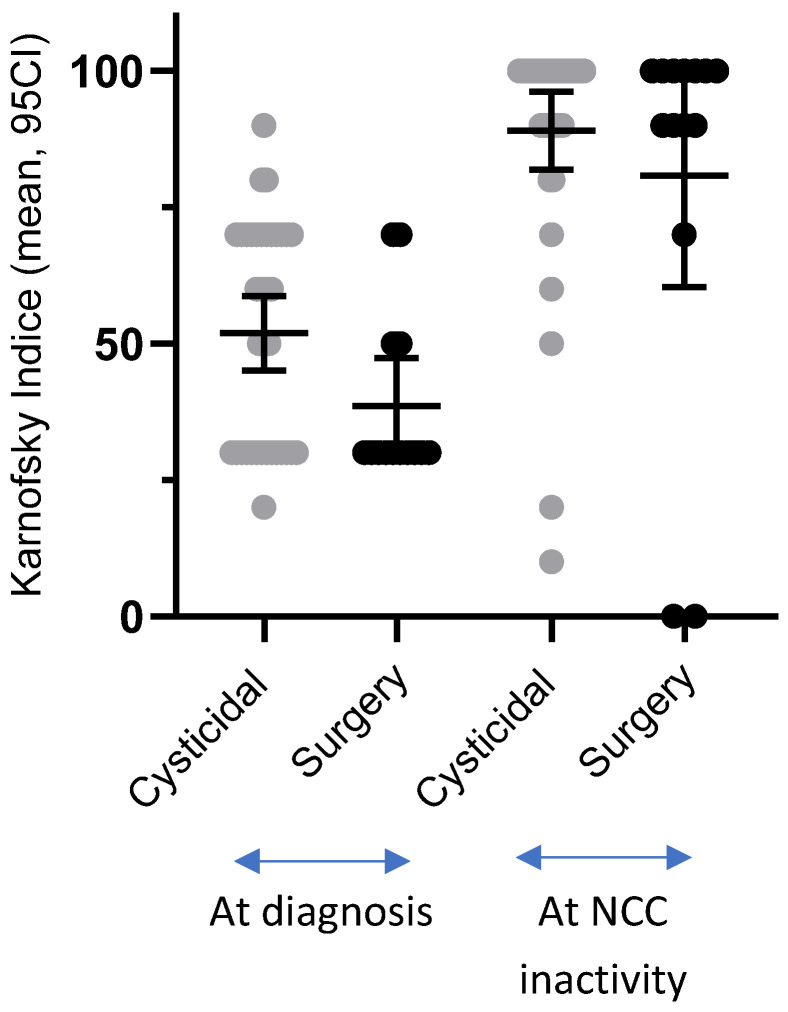
Changes in Karnofsky Index from diagnosis to disease inactivity in patients treated with cysticidal therapy or surgery.

At the time of inactivity, most patients in both groups were asymptomatic (64.86% in the cysticidal group and 64.28% in the surgical group, *p* = 1, Table 6). Their functionality (KI), which was reduced at the time of diagnosis in both groups, but more notably in the surgical group (*p* = 0.04), had increased significantly when disease inactivity was achieved. This was true in both groups, with no significant difference between them (*p* = 0.513). The individual evolution of KI values and the dispersion of functional outcomes in both treatment groups are illustrated in Figure 4.

Two patients (14.3%) died from nosocomial infections during follow-up, all of whom belonged to the surgical group.

## 4. Discussion

### 4.1. Comparative Effectiveness of Cysticidal Therapy and Surgical Management

In this retrospective study, we compared clinical and functional outcomes of cysticidal therapy and surgical management in patients with intraventricular NCC. Rather than demonstrating superiority of one strategy over the other, our findings suggest that cysticidal therapy can achieve long-term disease inactivity and functional outcomes comparable to surgical treatment, provided that intracranial hypertension is adequately managed. Although the proportion of inactive cases at six months was significantly higher in the surgical group, this difference did not persist over time, and patient functionality, as assessed by the Karnofsky Index at the time of disease inactivity, was good in most cases and comparable between groups.

Two deaths occurred in the surgical group, both related to nosocomial infections during prolonged hospitalizations rather than to the surgical procedure itself or to cysticidal treatment. Although the small number of events precludes any formal comparison between groups, this observation underscores the vulnerability of patients requiring complex neurosurgical management and extended inpatient care, particularly in resource-limited settings.

It should also be noted that most patients with intraventricular NCC also have vesicular cysts in other brain compartments, mainly in the subarachnoid space. In such cases, additional antiparasitic treatment will be necessary, even if surgery has been chosen for the management of ventricular cysts.

### 4.2. Current Recommendations and Ongoing Controversy in Intraventricular NCC Management

The management of intraventricular NCC remains a controversial issue. According to the most recent guidelines [6], the recommendations are as follows: for lateral and third ventricle NCC, neuroendoscopic removal is advised; for fourth ventricle NCC, surgical management is recommended; and for adherent intraventricular cysts, only management of hydrocephalus is indicated. The use of antiparasitic drugs is recommended only after shunt insertion when surgical removal of cysts is not feasible, but not after successful surgical removal of intraventricular cysts [8]. Notably, the strength of these recommendations is considered weak.

It is evident that endoscopic management represents an excellent therapeutic option in specialized centers. Our results support this view, as do those of a recent meta-analysis evaluating this approach [21]. However, as highlighted in the Introduction, endemic countries are often resource-limited, and both their medical infrastructure and the level of specialization of healthcare personnel are frequently insufficient to support the widespread use of endoscopy. Excluding cysticidal therapy as a treatment option in such contexts could therefore have negative consequences by promoting the use of endoscopy under suboptimal conditions.

In this setting, the results of our study are of particular interest and support cysticidal therapy as a reasonable initial management strategy, even when endoscopic removal could be considered. Our findings are consistent with several previously published case reports and case series [11,20,22,23].

### 4.3. Effectiveness of Cysticidal Therapy in Ventricular NCC

Our findings suggest that cysticidal therapy may be particularly effective in patients with ventricular cysts. Although direct quantitative comparisons across studies should be interpreted with caution, published series of subarachnoid NCC have reported lower rates of complete response after a single cysticidal cycle [9]. This difference is not unexpected, given that the ventricular space is much smaller than the subarachnoid compartment [24], limiting parasite growth, an important factor associated with treatment success [9]. Moreover, because the ventricular space is smaller, cysts come into close contact with ependymal cells much earlier, potentially facilitating a faster immune response and earlier cyst degeneration [25].

### 4.4. Surgical Management of Intracranial Hypertension

Our results highlight the high frequency of intracranial hypertension requiring surgical management in these patients. In the cysticidal group, 17 patients (45.9%) required surgery as an initial procedure, and 6 additional patients during follow-up, whereas in the surgical group, all patients required such treatment from the outset.

The type of procedure differed significantly between groups: in all patients from the cysticidal group, a ventriculoperitoneal shunt (VPS) was placed, while in most patients undergoing surgical management, a third ventriculostomy was performed. This difference is expected, as one of the advantages of extractive endoscopy is the possibility of performing a third ventriculostomy during the same surgical procedure.

Although VPS placement was historically associated with poor outcomes in NCC [26], results have markedly improved, and this procedure can now be considered a very good therapeutic option [2]. Advances such as improved diagnosis of extraparenchymal NCC through 3D MRI sequences [27,28] and the systematic use of corticosteroids to prevent inflammatory complications [2] have likely contributed to these favorable results. Experiences with endoscopic third ventriculostomy are more recent, but several series using a combined approach—extractive endoscopy plus third ventriculostomy—have reported very good outcomes, comparable to those reported here [21,29]. A recent meta-analysis comparing the efficacy and safety of endoscopic third ventriculostomy versus VPS in obstructive hydrocephalus reported similar operative success, but a significantly higher incidence of postoperative infection and blockage in the VPS group [30].

During follow-up, 11 patients required reintervention for VPS revision or replacement, and 6 patients required VPS placement. These repeat procedures were mainly performed in the group initially treated with cysticidal drugs (15/37, 40.5%). Patients should be informed of the risk and the need to consult the hospital if symptoms reappear. However, it is important to note that 14 patients in the cysticidal group (37.8%) did not require any surgical management of intracranial hypertension.

### 4.5. Economic and Contextual Considerations

Although a formal cost analysis was beyond the scope of this retrospective study, the economic implications of treatment choice for intraventricular NCC warrant consideration. Surgical management, particularly endoscopic removal, requires specialized equipment and trained neurosurgical teams, which may increase healthcare costs. Cysticidal therapy, while based on widely available antiparasitic drugs, often requires hospitalization during the treatment course, repeated imaging studies, additional treatment cycles in some patients, and surgical interventions for the management of hydrocephalus.

Overall, the economic impact of each strategy appears to be highly context-dependent and closely related to disease severity, the need for intracranial pressure management, and the availability of specialized neurosurgical resources. Prospective studies incorporating standardized cost analyses are needed to better inform treatment selection in endemic, resource-limited settings.

### 4.6. Study Limitations

This study has limitations inherent to its retrospective design. Treatment allocation was not randomized and depended on clinical judgment and available resources, which may have introduced selection bias. Follow-up duration was heterogeneous and not protocolized, although all patients were followed until radiological disease inactivity was achieved. Additionally, the relatively small sample size limited subgroup analyses. Prospective, randomized, multicenter studies would be required to strengthen the robustness of these findings.

## 5. Conclusions

In conclusion, both cysticidal therapy and endoscopic removal were able to achieve long-term disease inactivity and favorable functional outcomes in patients with intraventricular NCC. While surgical intervention was associated with faster parasite clearance, long-term functional outcomes were comparable between treatment strategies. Across both approaches, effective management depended largely on appropriate control of intracranial hypertension, underscoring the central role of cerebrospinal fluid diversion in patient outcomes. These results support a patient-centered, context-adapted therapeutic approach and highlight the need for prospective studies to better define evidence-based criteria for treatment selection.

## Figures and Tables

**Table 1:** Patient eligibility criteria and treatment allocation in the study.

	Criteria
Inclusion criteria	Age ≥ 18 years; definitive diagnosis of NCC according to accepted criteria [14,15]; intraventricular localization; available clinical and neuroimaging follow-up until disease inactivity; complete medical records
Exclusion criteria	Age < 18 years; incomplete clinical or imaging data; absence of follow-up until disease inactivity; alternative diagnosis
Treatment allocation	Retrospective, non-randomized. Initial treatment (cysticidal vs. surgical) was determined by the treating physicians based on ventricular location and the availability of neuroendoscopic expertise and equipment.

**Table 2:** Cysticidal and adjunctive pharmacological regimens used in the study.

Treatment Component	Regimen	Timing
Albendazole (ABZ)	30 mg/kg/day, orally, divided into two doses for 10 days	Initiated after stabilization of ICH. Used as initial therapy in the cysticidal group and administered postoperatively in selected surgical cases
Corticosteroids	Dexamethasone 24 mg/day (8 mg three times daily), with gradual tapering	Started 2–5 days prior to and continued concomitantly with cysticidal therapy
Repeat cysticidal cycles	One or two additional 10-day ABZ cycles (same dose) when required. Combined ABZ and praziquantel (PZQ, 50 mg/kg/day for 10 days) was administered in one patient.	Administered at least 6 months after the initial cycle, based on persistence of active cysts on MRI. *

* Treatment efficacy was routinely assessed by MRI approximately 6 months after cysticidal therapy. In some patients, follow-up imaging was delayed due to travel restrictions during the COVID-19 pandemic or logistical and economic constraints.

**Table 3:** General characteristics at diagnosis of patients included in the 2 groups.

	Cysticidal Drugs (n = 37)	Neurosurgery (n = 14)	*p*
Sex (feminine) *	22 (59.45)	7 (50)	0.752
Previous diagnosis of SA NCC *	3 (8.1)	2 (14.28)	0.606
At diagnosis of ventricular neurocysticercosis			
Age (mean ± SD)	36.35 ± 10.76	43.14 ± 12.01	0.0518
Symptoms *			
Without	1 (2.7)	0 (0)	1
Headache	6 (16.21)	1 (7.1)	0.657
ICH	29 (78.37)	13 (92.85)	0.413
Epileptic seizure	8 (21.62)	4 (28.57)	0.714
Other (hearing loss, vertigo, visual disturbances, vomiting)	8 (21.62)	4 (28.57)	0.714
Location of IV cysts *			
Lateral ventricles	11 (29.72)	2 (14.28)	0.472
Third ventricle	3 (8.1)	2 (14.28)	0.606
Fourth ventricle	20 (54.05)	7 (50)	1
Several locations	3 (8.1)	3 (21.42)	0.327
Degenerating stage of IV cysts *			
Vesicular	30 (81.08)	11 (78.57)	1
Colloidal	7 (18.91)	3 (21.42)	1
Number of IV cysts (single) *	20 (54.05)	5 (35.71)	0.348
Presence of parasites in other CNS locations *	27 (72.97)	7 (50)	0.183
Parenchyma	7 (25.92)	1 (14.28)	0.418
Subarachnoid	15 (55.55)	6 (85.71)	1
Mixed (parenchyma + subarachnoid)	5 (18.51)	0 (0)	0.305
Vesicular stage	22 (81.48)	5 (71.42)	0.208

* Data is presented as n (%).

**Table 4:** Treatment administrated.

Cysticidal Drugs (n = 37)	Neurosurgery (n = 14)
Initial treatment	
One ABZ cycle (20, 54.05%)	Endoscopy + surgical management of ICH (7, 50%)
One ABZ cycle + Surgical management of ICH (17, 45.94%) (DVP all)	Suboccipital craniotomy + Surgical management of ICH (2, 14.28%)
	Endoscopy + surgical management of ICH + cysticidal drugs (5, 35.71%)
Inactivity after initial treatment	
25 (67.56%)	11 (78.57%)
Complementary treatment in patients with inactive disease after initial treatment	
Re-intervention ICH (n = 9)	Re-intervention ICH (n = 1)
Complementary treatments in patients with active disease after initial treatment	
N = 12 (32.43%)	N = 3 (21.42%)
Another ABZ cycle (5, 41.66%)	Another ABZ cycle (1, 33.33%)
Another ABZ cycle + ICH surgical management (3, 25%)	Two other ABZ + PZQ cycles (1, 33.33%)
Another ABZ cycle + ICH surgical management + endoscopy (1, 8.3%)	Two other ABZ cycles + ICH surgical management (1, 33.33%)
Two other ABZ cycles (1, 8.3%)	
Two other ABZ cycles + ICH surgical management (2, 16.66%)	

**Table 5:** Type of surgical management of intracranial hypertension (ICH).

Cysticidal Drugs	Neurosurgery
Initial treatment of ICH (n = 17)	Initial treatment of ICH (n = 14)
VPS (n = 17, 100%)	VPS (n = 4, 28.57%) Endoscopic third ventriculostomy (n = 8, 57.14%) Both management (n = 2, 14.28%)
Complementary treatments	
VPS Re-intervention (n = 9, 52.94%) New colocation (n = 6)	VPS Re-intervention (n = 2, 14.28%)
Patients without surgical intervention of ICH during disease	
N = 14 (37.83%)	N = 0 (0%)

**Table 6:** Evolution of the two groups of patients.

	Cysticidal Drugs (n = 37)	Neurosurgery (n = 14)	*p*
Time to inactivity *			
<6 months	13 (35.13)	11 (78.57)	0.010
[6 months–1 year]	6 (16.21)	1 (7.14)	0.657
[1–2 years]	7 (18.91)	1 (7.14)	0.418
[2–3 years]	3 (8.1)	0 (0)	0.552
>3 years	8 (21.62)	1 (7.14)	0.413
Neurological symptoms at inactivity *			
Without	24 (64.86)	9 (64.28)	1
Headache	6 (16.21)	0 (0)	0.170
Epileptic seizure	3 (8.1)	1 (7.14)	1
Other (cognition, equilibrium, motor)	4 (10.81)	2 (14.28)	0.661
Karnofsky at diagnosis [CI 95%]	51.89 [45.1–58.7]	38.57 [29.8–47.3]	0.0424
Karnofsky at inactivity [CI 95%]	88.92 [81.8–96.1]	80.71 [60.4–100]	0.5135
*p*	<0.0001	0.0007	
Deaths at follow-up *	0 (0)	2 (14.28)	0.071

* Data is presented as n (%).

## Data Availability

Data supporting the findings of this study are available from the corresponding author upon reasonable request and subject to review.

## Abbreviations

The following abbreviations are used in this manuscript:

NCC	Neurocysticercosis
IV	Intraventricular
KI	Karnofsky Index
ABZ	Albendazole
PZQ	Praziquantel
ICH	Intracranial Hypertension
INNNMVS	Instituto Nacional de Neurología y Neurocirugía Manuel Velasco Suárez
MRI	Magnetic Resonance Imaging
SGPR	Spoiled Gradient Echo
FLAIR	Fluid-attenuated inversion recovery
3D	Three-dimensional
COVID	Coronavirus Disease
VPS	Ventriculoperitoneal shunt
SD	Standard deviation
SA	Subarachnoid
CI	Confidence Interval

## References

[1] Pineda-Reyes R., White A.C. (2022). Neurocysticercosis: An update on diagnosis, treatment, and prevention. Curr. Opin. Infect. Dis..

[2] Cruz L., Pacheco E., Soto W., Cong R., Suastegui R., Moreno-Jimenez S., Fleury A. (2023). Neurocysticercosis and hydrocephalus: The value of ventriculoperitoneal shunting in its management. Trans. R. Soc. Trop. Med. Hyg..

[3] Hamamoto Filho P.T., Rodríguez-Rivas R., Fleury A. (2022). Neurocysticercosis: A Review into Treatment Options, Indications, and Their Efficacy. Res. Rep. Trop. Med..

[4] Escobedo F., Penagos P., Rodriguez J., Sotelo J. (1987). Albendazole therapy for neurocysticercosis. Arch. Intern. Med..

[5] Sotelo J., Escobedo F., Rodriguez-Carbajal J., Torres B., Rubio-Donnadieu F. (1984). Therapy of parenchymal brain cysticercosis with praziquantel. N. Engl. J. Med..

[6] White A.C., Coyle C.M., Rajshekhar V., Singh G., Hauser W.A., Mohanty A., Garcia H.H., Nash T.E. (2018). Diagnosis and Treatment of Neurocysticercosis: 2017 Clinical Practice Guidelines by the Infectious Diseases Society of America (IDSA) and the American Society of Tropical Medicine and Hygiene (ASTMH). Am. J. Trop. Med. Hyg..

[7] Nash T.E., O’Connell E.M., Hammoud D.A., Wetzler L., Ware J.M., Mahanty S. (2020). Natural History of Treated Subarachnoid Neurocysticercosis. Am. J. Trop. Med. Hyg..

[8] Göngora-Rivera F., Soto-Hernández J.L., González Esquivel D., Cook H.J., Márquez-Caraveo C., Hernández Dávila R., Santos-Zambrano J. (2006). Albendazole trial at 15 or 30 mg/kg/day for subarachnoid and intraventricular cysticercosis. Neurology.

[9] Osorio R., Carrillo-Mezo R., Romo M.L., Toledo A., Matus C., González-Hernández I., Jung H., Fleury A. (2019). Factors Associated With Cysticidal Treatment Response in Extraparenchymal Neurocysticercosis. J. Clin. Pharmacol..

[10] Martinez H.R., Rangel-Guerra R., Arredondo-Estrada J.H., Marfil A., Onofre J. (1995). Medical and surgical treatment in neurocysticercosis a magnetic resonance study of 161 cases. J. Neurol. Sci..

[11] Proaño J.V., Torres-Corzo J., Rodríguez-Della Vecchia R., Guizar-Sahagun G., Rangel-Castilla L. (2009). Intraventricular and subarachnoid basal cisterns neurocysticercosis: A comparative study between traditional treatment versus neuroendoscopic surgery. Childs Nerv. Syst..

[12] Serpa J.A., Graviss E.A., Kass J.S., White A.C. (2011). Neurocysticercosis in Houston, Texas: An update. Medicine.

[13] Nash T.E., Ware J.M., Mahanty S. (2018). Intraventricular Neurocysticercosis: Experience and Long-Term Outcome from a Tertiary Referral Center in the United States. Am. J. Trop. Med. Hyg..

[14] Carpio A., Fleury A., Romo M.L., Abraham R., Fandiño J., Durán J.C., Cárdenas G., Moncayo J., Leite Rodrigues C., San-Juan D. (2016). New diagnostic criteria for neurocysticercosis: Reliability and validity. Ann. Neurol..

[15] Del Brutto O.H., Nash T.E., White A.C., Rajshekhar V., Wilkins P.P., Singh G., Vasquez C.M., Salgado P., Gilman R.H., Garcia H.H. (2017). Revised diagnostic criteria for neurocysticercosis. J. Neurol. Sci..

[16] Mont’Alverne Filho F.E., Machado Ldos R., Lucato L.T., Leite C.C. (2011). The role of 3D volumetric MR sequences in diagnosing intraventricular neurocysticercosis: Preliminar results. Arq. Neuropsiquiatr..

[17] Karnofsky D.A., Abelmann W.H., Craver L.F., Burchenal J.H. (1948). The use of the nitrogen mustards in the palliative treatment of carcinoma with particular reference to bronchogenic carcinoma. Cancer.

[18] Torres-Corzo J.G., Tapia-Pérez J.H., Vecchia R.R.D., Chalita-Williams J.C., Sánchez-Aguilar M., Sánchez-Rodríguez J.J. (2010). Endoscopic management of hydrocephalus due to neurocysticercosis. Clin. Neurol. Neurosurg..

[19] Crooks V., Waller S., Smith T., Hahn T.J. (1991). The use of the Karnofsky Performance Scale in determining outcomes and risk in geriatric outpatients. J. Gerontol..

[20] Zhu L., Weng X., Shi Y., Pan X., Mo L. (2002). CSF-VP shunt placement and albendazole therapy for cerebral cysticercosis. Chin. Med. J..

[21] Mendieta-Barrera C.D., Punukollu A., Rios-Hurtado C., De Nigris Vasconcellos F., Garcia-Torrico F., Salolin-Vargas V.P., Mamani-Julian K., Valderrama C.E.V., Rivera-Hurtado L., Ballesteros-Herrera D. (2025). Neuroendoscopic management of intraventricular neurocysticercosis: A systematic review and meta-analysis. Clin. Neurol. Neurosurg..

[22] Hajek J., Keystone J. (2009). Intraventricular neurocysticercosis managed with albendazole and dexamethasone. Can. J. Neurol. Sci..

[23] Mesa E., Ruprecht V., Nguyen M.C., Casadesus D. (2023). Vertigo: An Atypical Presentation of Neurocysticercosis Successfully Treated With Albendazole. Cureus.

[24] Sakka L., Coll G., Chazal J. (2011). Anatomy and physiology of cerebrospinal fluid. Eur. Ann. Otorhinolaryngol. Head Neck Dis..

[25] Xie S., Li F. (2024). Ependymal cells: Roles in central nervous system infections and therapeutic application. J. Neuroinflamm..

[26] Sotelo J., Marin C. (1987). Hydrocephalus secondary to cysticercotic arachnoiditis: A long-term follow-up review of 92 cases. J. Neurosurg..

[27] Govindappa S.S., Narayanan J.P., Krishnamoorthy V.M., Shastry C.H.S., Balasubramaniam A., Krishna S.S. (2000). Improved Detection of Intraventricular Cysticercal Cysts with the Use of Three-dimensional Constructive Interference in Steady State MR Sequences. AJNR Am. J. Neuroradiol..

[28] Carrillo Mezo R., Lara García J., Arroyo M., Fleury A. (2015). Relevance of 3D magnetic resonance imaging sequences in diagnosing basal subarachnoid neurocysticercosis. Acta Trop..

[29] De Marco R., Lacatena F., Cofano F., Garbossa D., Fiumefreddo A. (2024). A case-based review on the neuroendoscopic management of intraventricular and subarachnoid basal neurocysticercosis. Clin. Neurol. Neurosurg..

[30] Pasqualotto E., Schmidt P.H.S., Ferreira R.O.M., Chavez M.P., Da Silva F.F.S. (2023). Endoscopic Third Ventriculostomy versus Ventriculoperitoneal Shunt in Patients with Obstructive Hydrocephalus: An Updated Systematic Review and Meta-Analysis. Asian J. Neurosurg..

